# Imputing missing covariate values for the Cox model

**DOI:** 10.1002/sim.3618

**Published:** 2009-05-19

**Authors:** Ian R White, Patrick Royston

**Affiliations:** 1 MRC Biostatistics Unit, Institute of Public HealthRobinson Way, Cambridge CB2 0SR, U.K.; 2 MRC Clinical Trials UnitCancer Group, London, U.K.

**Keywords:** missing data, missing covariates, multiple imputation, proportional hazards model

## Abstract

Multiple imputation is commonly used to impute missing data, and is typically more efficient than complete cases analysis in regression analysis when covariates have missing values. Imputation may be performed using a regression model for the incomplete covariates on other covariates and, importantly, on the outcome. With a survival outcome, it is a common practice to use the event indicator *D* and the log of the observed event or censoring time *T* in the imputation model, but the rationale is not clear.

We assume that the survival outcome follows a proportional hazards model given covariates *X* and *Z*. We show that a suitable model for imputing binary or Normal *X* is a logistic or linear regression on the event indicator *D*, the cumulative baseline hazard *H*_0_(*T*), and the other covariates *Z*. This result is exact in the case of a single binary covariate; in other cases, it is approximately valid for small covariate effects and/or small cumulative incidence. If we do not know *H*_0_(*T*), we approximate it by the Nelson–Aalen estimator of *H*(*T*) or estimate it by Cox regression.

We compare the methods using simulation studies. We find that using log *T* biases covariate-outcome associations towards the null, while the new methods have lower bias. Overall, we recommend including the event indicator and the Nelson–Aalen estimator of *H*(*T*) in the imputation model. Copyright © 2009 John Wiley & Sons, Ltd.

## 1. INTRODUCTION

Multiple imputation (MI) [1] is commonly used to perform statistical inference in the presence of missing data. Unlike simpler imputation methods, it can yield inferences that accurately reflect the uncertainty due to the missing data. MI is typically more efficient than complete cases analysis when covariates have missing values. Implementations in Stata [2, 3], SAS [4] and R [5] have led to its widespread use.

The main difficulty in MI lies in appropriately performing the imputations. We initially focus on the case where only one variable is incomplete. When all variables are discrete, imputation may be performed within cells defined by the complete variables [6]. More commonly, a regression model is used, termed the *imputation model*, as opposed to the *analysis model* whose regression coefficients are of substantive interest. The choice of variables in the imputation model is crucial: in particular, any association to be assessed in the analysis model must be allowed for in the imputation model [7, 8], for otherwise bias towards the null is likely. When the incomplete data are covariates in the analysis model, the analysis model outcome must be used to predict the missing covariate values. Although this practice may seem counter-intuitive, it is in fact essential [9].

In this paper, we consider analysis of a (typically censored) survival outcome in relation to one or more incomplete covariates. Each imputation model involves regression of an incomplete covariate on the other covariates and on survival. It is important to find the right way to include the survival outcome in this imputation model because, otherwise, the association between the covariate and survival is likely to be diluted. An influential paper on the practical use of MI used the event indicator *D*, the observed event or censoring time *T* and the log of *T* as predictors in the imputation model [10], while other authors have used just *D* and the log of *T* [11] or just *D* and *T* [12]. However, it is not clear which procedure is correct: for example, is the log transformation appropriate, and should an interaction between *D* and *T* be included in the imputation model?

The aim of this paper is to develop a more principled approach to including a survival outcome in an imputation model. We focus on the case where the outcome is assumed to follow a proportional hazards model, although other cases are mentioned in the discussion. The methods will be presented for the case of a single incomplete variable, but the same issues arise with multiple incomplete variables, whether they are handled by fitting a multivariate normal distribution to the data via MCMC [4, 8, 13], by sequential application of regression imputation to monotonic missing data [4], or by iterative application of regression imputation to non-mono tonic missing data (multiple imputation by chained equations, MICE) [10]. We assume throughout that the data are missing at random or missing completely at random [14], and that censoring is non-informative.

In Section 2, we present a motivating data set in renal cancer. In Section 3, we explore the imputation model algebraically. With a single binary covariate, we show that the correct imputation model is a logistic regression on the event indicator *D* and the cumulative baseline hazard *H*_0_(*T*) at the time of event/censoring. In a more general multivariable situation with a binary or Normally distributed covariate, we show that the corresponding result is approximately valid when covariate effects are small and/or cumulative incidence is small. We also propose two ways to approximate the unknown *H*_0_(*T*). In Section 4, we report simulation studies comparing the methods in the univariate and bivariate cases. In Section 5, we apply the different methods in fitting a prognostic model in renal cancer. We end with a discussion and recommendations in Section 6.

## 2. EXAMPLE: RENAL CANCER DATA

The MRC RE01 study was a randomized controlled trial comparing treatment with interferon-α (IFN) with best supportive care and hormone treatment with medroxyprogesterone acetate (control) in patients with metastatic renal carcinoma. The study recruited 350 patients between 1992 and 1997 [15].

In this illustrative analysis, we attempt to build a prognostic model using the erythrocyte sedimentation rate (ESR), a variable that was only collected for half of the patients. ESR was broadly missing at hospital level: that is, its measurement appears to have been largely a matter of hospital policy, and it appears to be approximately MCAR. We exclude three patients with no follow-up. A number of possible prognostic variables were available, from which five were selected by analysis of the patients with observed ESR. The variables are listed in Table I. Analysis by multivariable fractional polynomials [16] suggested that wcc and t_mt should be entered into the analysis model as wcc" 3 and log (t_mt + 1), respectively.

**Table I:** Summary of data from the MRC RE01 study (*n* = 347).

Variable	Code	Mean	SD	Per cent missing
Erythrocyte sedimentation rate	esr	49.6	35.1	51.3
Haemoglobin	haem	12.3	1.9	6.6
White cell count	wcc	8.7	4.1	6.6
Days from metastasis to randomization	t_mt	129	421	0.3
		*Value*	*per cent*	
WHO performance status	who	0	27	0
		1	48	
		2	24	
Treatment with IFN	trt	control	50	0
		IFN	50	

Because of the large number of missing values of ESR, complete cases analysis uses less than half the data set. However, the rest of the data set carries information about the associations between the other covariates and the outcome, so it is sensible to use MI for the analysis of these data. We will use the data to compare different ways to incorporate the outcome in the imputation model.

## 3. METHODS

### 3.1. Multiple imputation

We briefly describe MI for a single incomplete variable *X*, a vector of complete variables *Z* and complete outcome *Y*. We assume that we have an *imputation model p*(*X*|*Y*, *Z*; α) parameterized by α. Formally, MI involves drawing values of the missing data *X*^mis^ from the predictive distribution *p*(*X*^mis^|*X*^obs^, *Y, Z*) = ∫ *p* (*X*^mis^|*X*^obs^, *Y*, *Z*; α)*p*(α|*X*^ohs^, *Y*, *Z*)dα, where *p*(α|*X*^obs^, *Y*, *Z*) is the Bayesian posterior distribution of α [1]. In practice, this may be achieved (with implicit vague priors) by (1) fitting the model *p*(*X*|*Y*, *Z*; α) to the cases with observed *X*, yielding an estimate (typically an MLE) 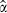 with estimated variance–covariance matrix *S*_α_; (2) drawing a value of α, α*, say, from its posterior, perhaps approximated as 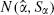; and (3) drawing values of *X*^mis^ from *p*(*X*|*Y, Z*;α*)[6].

Where some of the *Z* variables are also incomplete, the method of MI by chained equations (MICE) [10] starts by filling in missing values arbitrarily, then applies the above univariate method for each incomplete variable in turn, using the current imputed values of *Z* when drawing new values of *X*, and vice versa. The procedure is iterated until convergence, which often requires fewer than 10 cycles [2]. An alternative non-iterative procedure is available if the data are monotonically missing [8].

Once imputed data sets have been created, analysis is performed on each data set separately. Let *Q_r_* be the point estimate of a (scalar or vector) parameter of interest for the *r*th imputed data set (*r* = 1, …, *m*) with variance–covariance matrix *U_r_*. These values are then combined by Rubin's rules [1]: the overall point estimate is 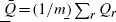 with variance *Ū* + (1 + 1/*m*)*B*, where *Ū* = (1/*m*) Σ_*r*_ *U_r_* and 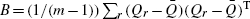. Tests and confidence intervals for a scalar parameter are constructed using a *t*-distribution with degrees of freedom given by Rubin's formula [1] or an alternative [17].

### 3.2. Conditional distribution of covariates

We now focus on the case of a survival outcome *T* with event indicator *D* (1 for events, 0 for censored observations). We assume that the outcome follows the Cox proportional hazards model *h*(*t*|*X, Z*) = *h*_0_(*t*)exp(β_*X*_ *X* + β_*Z*_ *Z*) where again *X* is incomplete and *Z* is complete. We also need an ‘exposure model’ *p*(*X*|*Z*;ζ) in order to allow for the incomplete *X*.

In the appendix, we prove a number of exact and approximate results about the imputation model *p*(*X*|*T, D, Z*) in terms of the model parameters θ = (ζ, β_*X*_, β_*Z*_, *h*_0_(.)). These results are used to motivate regression models *p*(*X*|*T, D, Z*; α), where the parameter α is some function of θ. In practice, we do not know θ, but we can estimate the parameters α directly from the complete cases. Therefore, the models below are stated in terms of the unknown parameters α, which typically differ across different models.

First, with binary *X* and no *Z*, we have


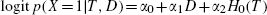


where *H*_0_(*T*) is the cumulative baseline hazard 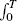 *h*_0_(*t*)d*t*. In other words, the missing *X* may be imputed by fitting a logistic regression of *X* on *D* and *H*_0_(*T*) to the complete cases.

Second, with binary *X* and binary or categorical *Z*, if we take the most general exposure model logit *p*(*X* = 1|*Z*) = ζ_*Z*_, then we get


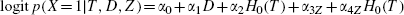


where terms such as α_3*Z*_ represent a set of dummy variables with their coefficients.

In other cases we can only obtain approximate results. For binary *X* with more general (possibly vector-valued) *Z*, we make a Taylor series approximation for exp(β_*Z*_*Z*) that is valid when β_*Z*_*Z* has small variance. Using the exposure model logit *p*(*X* = 1|*Z*) = ζ_0_ + ζ_1_*Z* gives


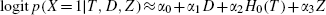


and the addition of an interaction term α_4_ *H*_0_(*T*)*Z* improves the accuracy of the approximation. Further, if the user believed that a particular transformation of *Z* was needed for predicting *X*, then this transformation should be entered in the imputation model.

For Normal *X*, we make a fuller Taylor series approximation for exp(β_*X*_*X* + β_*Z*_*Z*) that is valid when β_*X*_*X* + β_*Z*_*Z* has small variance. Using the exposure model *X*|*Z*∼N(ζ_0_ + ζ_1_*Z*, sigma;^2^) gives


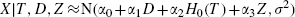


using a first-order approximation, and again the addition of an interaction term α_0_*H*_0_(*T*)*Z* improves the accuracy of the approximation. Equations (A7) and (A8) in the appendix suggest that departures from the above model will be most marked when both var(β_*X*_*X*) and 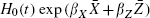 (roughly the overall cumulative hazard at the event time *T*) are large.

### 3.3. A small empirical investigation

**Figure 1:**
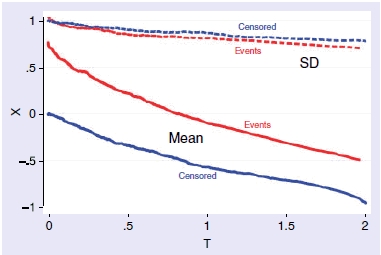
Smoothed mean and SD of *X*|*T, D* with β_*X*_ = 0.7, *h*_0_(*t*) = 1.

We explored the distribution of *X*|*T, D* empirically using 100000 simulated data points and the model described above with standard Normal *X*, β_*X*_ = 0.7, *h*_0_(*t*) = 1 (so *H*_0_(*t*) = *t*) and censoring times uniformly distributed on [0,2]. Figure 1 shows smoothed graphs of the conditional mean and standard deviation, *E*[*X*|*T, D*] and SD(*X*|*T, D*). A linear regression on *D* and *H*_0_(*T*) would be shown by parallel straight lines for the mean and a constant SD. Some departures from linear regression are seen: the mean graphs are somewhat curved and converging, and the SD declines with *T*.

Taken together, these results suggest that logistic or linear regression of *X* on *D*, *H*_0_(*T*) and *Z* may be appropriate in many situations, and that including an interaction between *Z* and *H*_0_(*T*) may improve the approximation, but that the approximation will not work well in situations with strong covariate effects and large cumulative incidences.

### 3.4. Implementation

In practice, *H*_0_(*T*) is unknown and must be estimated. We consider three possible methods.

#### 3.4.1. Substantive knowledge

In many applications, the baseline hazard may be approximately known: for example, in following a cohort of healthy individuals over a small number of years, the baseline hazard could be assumed to be roughly constant. In this case it would be reasonable to assume *H*_0_(*T*) ∝ *T*. This may be a useful ‘off-the-shelf’ method.

#### 3.4.2. Nelson–Aalen method

When the covariate effects β_*X*_ and β_*Z*_ are small, we may approximate *H*_0_(*T*) ≈ *H*(*T*), which is easily estimated before imputation using the Nelson–Aalen estimator. It seems possible that this method will perform well for moderate sized β_*X*_ and β_*Z*_ because small errors in estimating *H*_0_(*T*) are unlikely to have much impact on the imputations.

#### 3.4.3. Cox method

We also propose estimating *H*_0_(*T*) iteratively: first, imputing *X* using the current estimate of *H*_0_(*T*), then fitting the Cox proportional hazards model to the data using the current values of the covariates *X, Z* and extracting a revised estimate of the baseline hazard function *H*_0_(*T*). This fits conveniently within the MICE algorithm: in each imputation cycle, as well as updating each incomplete variable in turn, we also update *H*_0_(*T*) by fitting the Cox model.

Because it is unlikely that *H*_0_(*T*) will change much from one iteration to the next, we also consider a less computationally intensive version in which *H*_0_(*T*) is updated only on the first *k* cycles. Here we will use *k* = 2.

#### 3.4.4. Theoretical properties

We note that the methods described in Sections 3.4.2 and 3.4.3 do not acknowledge the uncertainty in estimating *H*_0_(*T*). AS a result, they are not Bayesianly proper [1], so that standard errors may be too small and confidence intervals may be too narrow. However, we do allow for uncertainty in the coefficient of *H*_0_(*T*), so we do not expect any undercoverage to be important.

## 4. SIMULATION STUDY

We now present simulation studies to compare the methods introduced in Section 3. These are summarized in Table II. We first consider the simple case of binary or Normal *X* and no *Z*.

**Table II:** Models considered for imputing missing values of incomplete *X*.

Abbreviation	Description
NO-T	Regression of *X* on *Z*
LOGT	Regression of *X* on *Z, D* and log*t*
T	Regression of *X* on *Z, D* and *T* (appropriate if the shape κ=1)
T2	Regression of *X* on *Z, D* and *T*^2^ (appropriate if the shape κ = 2)
NA	Regression of *X* on *Z, D* and 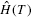 , the Nelson–Aalen estimator of *H(T)*
NA-INT	Regression of *X* on *Z, D*, 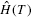 and *z* × 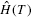
COX	Regression of *X* on *Z, D* and 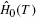 , where 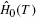 is estimated iteratively as described in Section 3.4.3
COX*	Same as COX, but with only two iterations used for 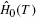 as described in Section 3.4.3

‘Regression’ means logistic regression for binary *X* and linear regression for Normal *X*.

### 4.1. One covariate: design of simulation study

The covariate *X* was either binary with *P*(*X* = 1) = π*X* or standard Normal, so that its standard deviation 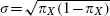 or 1, respectively. *X* was missing completely at random with probability π_*M*_. Survival times were drawn from a Weibull distribution *h_T_*(*t*) = λ_*T*_ κ*t*^κ−1^ exp (β_*X*_*X*). Random censoring times were drawn from a Weibull distribution with the same shape parameter, *h_C_*(*t*) = λ_*C*_ κ*t*^κ−1^. The parameter values used were π_*M*_ = 0.5; π_*M*_ = 0.5; π_*T*_ =0.002; κ = 1; β_*X*_σ = 0,0.5,1; and λ_*C*_ =0.002 (corresponding to approximately 50 per cent censoring). When β_*X*_ ≠ 0, the sample size *n* was chosen to give 90 per cent power to detect a significant association between binary *X* and survival at the 5 per cent level, using Collett's formula [18]. For Normal *X*, the sample size was chosen to be the same as for binary *X* with the same value of β_*X*_σ. When β_*X*_ = 0, the sample size was chosen to be the same as that for β_*X*_σ = 0.5.

In sensitivity analyses, one parameter was varied at a time: ‘High censoring’, λ_*C*_ = 0.01 (corresponding to approximately 83 per cent censoring); ‘low missing’, π_*M*_ = 0.2; ‘shape 2’, κ = 2; and ‘administrative censoring’, censoring at a fixed time computed to give the same censored fraction as random censoring when *X* = 0.

The imputation methods described above were used to construct *m* = 10 imputed data sets. The analysis model was a Cox regression on *X*. Results from the imputed data sets were combined using Rubin's rules as described in Section 3.1. For comparison, we also analysed each simulated data set before introducing missing values (PERFECT) and using complete cases only (CC). In each case we estimated the bias and the empirical standard error of the point estimate; the relative error in the average model-based standard error, defined as its difference from the empirical standard error of the point estimate minus 1; the coverage of a nominal 95 per cent Normal-theory confidence interval; and the power of a Normal-theory 5 per cent significance test of the null hypothesis of β=0.

#### 4.1.1. One covariate: results for binary X

Table III shows the key results. NO-T is strongly biased towards the null, the proportionate bias equalling the proportion of missing data. LOGT is mildly biased (up to 10 per cent) towards the null. All other methods have no appreciable bias. All methods except NO-T have similar empirical standard errors (results not shown). NO-T has smaller empirical standard error as a result of its bias towards the null.

**Table III:** Simulation results for parameter β in univariate model with binary *X*.

	Settings		Analyses								
		
Model	β	*n*	PERFECT	CC	NO-T	LOGT	T	T2	NA	COX	COX*
*Bias*											
Base case*	0	336	0.00	−0.01	−0.01	−0.01	−0.01	−0.01	−0.01	−0.01	−0.01
	0.5	336	0.00	0.00	**−0.26**	**−0.06**	0.00	−0.02	0.00	0.00	0.00
	1	84	0.01	0.03	**−0.52**	**−0.08**	0.03	0.02	0.03	**0.04**	**0.04**
Shape 2	1	84	0.01	0.03	**−0.52**	**−0.08**	0.00	0.03	0.03	**0.04**	**0.04**
Admin cens†	1	84	0.01	0.03	**−0.51**	0.01	0.02	0.02	0.02	0.02	0.02
*Per cent error in model standard error*											
Base case	0	336	0	1	**71**	4	−1	0	−1	0	1
	0.5	336	−1	1	**73**	5	0	−1	0	0	0
	1	84	−2	−6	**74**	4	−2	−4	−2	−4	−2
Shape 2	1	84	−2	−6	**74**	4	0	−2	−2	−4	−2
Admin cens	1	84	−4	−1	**74**	−1	−2	−2	−2	0	0
*Power*											
Base case	0.5	336	92	65	**14**	**59**	65	**62**	65	64	64
	1	84	92	64	**12**	**57**	64	**60**	63	64	65
Shape 2	1	84	92	64	**12**	**57**	63	64	63	64	64
Admin cens	1	84	96	73	**17**	71	73	73	72	71	71

* Base case: π_*M*_ = 0.5, π_*X*_ = 0.5, λ_*T*_ =0.002, shape κ=1, random censoring with λ_*C*_ = 0.002.

† Administrative censoring.

Monte Carlo error: ≤0.016 for bias, ≤4 per cent for per cent error in model standard error, ≤1.6 per cent for power. Bold cells have bias greater than 0.03, more than 10 per cent error in model standard error or power more than 3 per cent worse than method T.

All methods except NO-T have model-based standard errors that compare well with the empirical standard errors. NO-T has a standard error that is up to 70 per cent too large. All methods except NO-T have coverage between 93 and 96 per cent in all cases, while coverage of NO-T varies from 73 to 100 per cent (results not shown).

Power was very low for NO-T, reduced by up to 6 per cent for LOGT and by up to 3 per cent for T2 (only when κ = 1), compared with other methods. Differences in power between other methods appear to be consistent with chance.

Results with ‘high censoring’ were very similar to the base case; results with ‘low missing’ showed weaker patterns than the base case; and with ‘Shape 2’ and ‘Administrative censoring’, results with β < 1 showed weaker patterns than those shown with β = 1.

Because of its poor properties, we do not consider NO-T in further simulation studies.

#### 4.1.2. One covariate: results for Normal X

The results in Table IV show that some methods, notably COX but also LOGT and T2 (when κ = 1), show small bias towards the null. (Note that when *n*=84 the PERFECT and CC methods show small-sample bias away from the null.) We did not explore precision because of the presence of bias. Coverage was 93–96 per cent for LOGT, T, T2 and NA, and 92–97 per cent for COX and COX* (results not shown). Power was greatest with T, NA and COX* methods. T2 had noticeably less power than T when κ=1, but was not superior when κ = 2.

**Table IV:** Simulation results for parameter β in univariate model with Normal *X*.

	Settings		Analyses							
		
Model	β	*n*	PERFECT	CC	LOGT	T	T2	NA	COX	COX*
*Bias*										
Base case*	0	336	0.00	0.00	0.00	0.00	0.00	0.00	0.00	0.00
	0.25	336	0.00	0.01	−0.02	0.00	−0.01	0.00	0.00	0.00
	0.5	84	0.02	**0.04**	**−0.04**	−0.03	**−0.06**	−0.02	**−0.08**	−0.03
Shape 2	0.5	84	0.02	**0.04**	**−0.04**	−0.02	−0.03	−0.02	**−0.08**	−0.02
Admin cens†	0.5	84	0.01	0.01	−0.03	−0.03	**−0.03**	−0.03	−0.03	−0.02
*Per cent error in model standard error*										
Base case	0	336	−2	−2	3	−1	1	−1	−1	0
	0.25	336	−3	−4	2	0	4	0	6	0
	0.5	84	0	−3	**11**	**10**	**14**	9	8	**10**
Shape 2	0.5	84	0	−3	**11**	9	**10**	9	8	**10**
Admin cens	0.5	84	−1	−5	1	1	2	1	2	1
*Power*										
Base case	0.25	336	89	62	**55**	60	**56**	60	59	60
	0.5	84	88	58	**47**	53	**49**	53	**43**	55
Shape 2	0.5	84	88	58	**47**	52	53	53	**43**	55
Admin cens	0.5	84	89	58	53	53	53	53	55	54

* Base case: π_*M*_ = 0.5, π_*X*_ = 0.5, λ_*T*_ = 0.002, shape κ=1, random censoring with λ_*C*_ = 0.002.

† Administrative censoring.

Monte Carlo error: ≤0.01 for bias, ≤5 per cent for per cent error in model standard error, ≤1.6 per cent for power. Bold cells have bias greater than 0.03, more than 10 per cent error in model standard error or power more than 3 per cent worse than method T.

We conclude that LOGT is somewhat suspect because of potential bias towards the null. All other methods considered are adequate, and T, NA and COX* may be the best. There is no gain from the extra computational burden in COX, which if anything performs worse than COX*.

### 4.2. Two covariates: design of simulation study

We next added in a complete covariate *Z*. We took *X* and *Z* to be standard Normal with correlation ρ. The analysis model was now *h_T_(t)*=λ_*T*_κ*t*^κ−1^ exp(β_*X*_*X* + β_*Z*_*Z*). We were especially interested in seeing what happens as β_*X*_ and β_*Z*_ get larger, since Section 3.2 suggested that this is where our approximations may break down.

We induced missing data in *X* only, using a MCAR mechanism as before. We took π_*M*_ = 0.5, λ_*T*_ = 0.002, κ = 1 and random censoring with λ_*C*_ = 0.002 in all simulations: these choices for π_*M*_ and λ_*C*_ were found in the univariate study to be most sensitive to different analysis methods. Further, we took all combinations of ρ = 0,0.5; β_*X*_ = 0,0.25,0.5; and β_*Z*_ = 0,0.25,0.5.

To explore how the missing data mechanism affects the results, we repeated the bivariate simulation under the MAR mechanism logit *P* (*M_X_* | *X, Z*) = *Z*, where *M_X_* indicates missingness of *X*: this yielded 50 per cent missing values. We did this in the case β_*X*_ = β_*Z*_ = 1 only.

We used all the methods proposed before, with the exception of NO-T, which had performed very poorly, and COX, which had not performed well enough to justify its computational burden in the univariate study. In addition, we introduced a modification of the NA method that includes the interaction of *Z* with *H(T)* in the imputation model: we call this method NA-INT.

#### 4.2.1. Two covariates: results

Results for 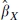 are given in Table V. We first consider the MCAR case. Bias towards the null increases with increasing values of β_*X*_, β_*Z*_ and ρ. It is worst for T2, being up to 20 per cent of the true value of β. Precision is not compared because of the presence of bias. Model-based standard errors are up to 17 per cent too high, with the discrepancy increasing with β_*X*_. Despite these problems, coverage was adequate (94–97 per cent) for all methods (results not shown). Power was greatest with T, NA, NA-INT and COX* methods, and worst for LOGT and T2. The NA-INT method performed very similarly to the NA method.

**Table V:** Simulation results for parameter β_*X*_ in bivariate model.

	Settings				Analyses							
		
	β_*X*_	β_*Z*_	ρ	*n*	PERFECT	CC	LOGT	T	T2	NA	NA-INT	COX*
*Bias*												
MCAR	0	0	0	336	0.00	0.00	0.00	0.00	0.00	0.00	0.00	0.00
	0	0	0.5	336	0.00	−0.01	0.00	−0.01	0.00	−0.01	0.00	0.00
	0	0.5	0	336	0.00	0.00	0.00	0.00	0.00	0.00	0.00	0.00
	0	0.5	0.5	336	−0.01	−0.01	0.00	0.00	0.00	0.00	−0.01	0.00
	0.5	0	0	84	0.01	**0.03**	**−0.05**	**−0.03**	**−0.06**	−0.03	**−0.04**	**−0.03**
	0.5	0	0.5	84	0.01	**0.03**	**−0.05**	**−0.04**	**−0.07**	**−0.03**	**−0.05**	**−0.04**
	0.5	0.5	0	84	0.00	0.03	**−0.06**	**−0.05**	**−0.09**	**−0.04**	**−0.05**	**−0.05**
	0.5	0.5	0.5	84	0.01	0.03	**−0.06**	**−0.07**	**−0.10**	**−0.05**	**−0.05**	**−0.07**
MAR	0.5	0.5	0	84	0.00	**0.04**	**−0.08**	**−0.09**	**−0.12**	**−0.07**	**−0.07**	**−0.08**
	0.5	0.5	0.5	84	0.01	**0.04**	**−0.09**	**−0.11**	**−0.14**	**−0.09**	**−0.08**	**−0.11**
*Per cent error in model standard error*												
MCAR	0	0	0	336	0	1	5	1	4	1	3	1
	0	0	0.5	336	0	4	8	4	7	4	6	5
	0	0.5	0	336	1	61	5	2	6	1	2	3
	0	0.5	0.5	336	2	4	8	7	**11**	6	7	9
	0.5	0	0	84	0	−6	8	7	**11**	5	6	9
	0.5	0	0.5	84	0	−5	9	7	**10**	6	5	8
	0.5	0.5	0	84	−2	−5	9	9	**14**	6	6	**11**
	0.5	0.5	0.5	84	−2	−2	**13**	**13**	**17**	**11**	10	**15**
MAR	0.5	0.5	0	84	−2	−9	**11**	**13**	**17**	**10**	7	**14**
	0.5	0.5	0.5	84	−2	−3	**15**	**19**	**24**	**16**	**12**	**18**
*Per cent power*												
MCAR	0.5	0	0	84	85	54	**44**	50	**46**	50	48	49
	0.5	0	0.5	84	74	42	36	39	36	39	38	40
	0.5	0.5	0	84	85	53	44	46	**41**	48	47	47
	0.5	0.5	0.5	84	73	43	32	34	**30**	35	37	34
MAR	0.5	0.5	0	84	85	49	38	39	**34**	40	40	41
	0.5	0.5	0.5	84	73	38	27	27	**22**	28	30	27

Monte Carlo error is ≤0.01 for bias, ≤3 per cent for per cent error in model standard error, ≤l.6 for power.

Bold cells have bias greater than 0.03, more than 10 per cent error in model standard error or power more than 3 per cent worse than method T.

Results for the MAR case show increased bias in 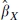, increased error in the model-based standard errors and decreased power, but the comparisons between methods are similar to the MCAR case.

Results for 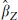 are given in Table VI. There was small bias away from the null when β_*X*_>0 and ρ=0.5 because the small bias in 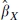 seen previously leads to residual confounding. Model-based standard errors were all accurate to within 10 per cent. Coverages ranged from 94 to 97 per cent (results not shown). Power was similar for all MI methods, but was substantially greater for MI than for CC.

**Table VI:** Simulation results for parameter β_*Z*_ in bivariate model.

	Settings				Analyses							
		
	β_*X*_	β_*Z*_	ρ	*n*	PERFECT	CC	LOGT	T	T2	NA	NA-INT	COX*
*Bias*												
MCAR	0	0	0	336	0.00	0.01	0.00	0.00	0.00	0.00	0.00	0.00
	0	0	0.5	336	0.00	0.01	0.00	0.00	0.00	0.00	0.00	0.00
	0	0.5	0	336	0.01	0.01	0.01	0.01	0.01	0.01	0.01	0.01
	0	0.5	0.5	336	0.01	0.02	0.01	0.01	0.01	0.01	0.01	0.01
	0.5	0	0	84	0.00	0.00	0.01	0.01	0.01	0.01	0.01	0.01
	0.5	0	0.5	84	0.00	0.01	0.03	0.02	**0.03**	0.02	**0.03**	0.02
	0.5	0.5	0	84	0.02	**0.03**	0.00	0.00	−0.01	0.00	0.02	0.00
	0.5	0.5	0.5	84	0.02	**0.04**	**0.03**	0.03	**0.04**	0.03	**0.04**	**0.03**
MAR	0.5	0.5	0	84	0.02	**0.04**	0.00	−0.01	−0.01	−0.01	0.01	−0.01
	0.5	0.5	0.5	84	0.02	**0.05**	**0.04**	**0.04**	**0.05**	**0.04**	**0.04**	**0.04**
*Per cent error in model standard error*												
MCAR	0	0	0	336	−2	1	−1	−1	−1	−1	−2	−1
	0	0	0.5	336	−2	1	2	1	2	1	1	1
	0	0.5	0	336	0	0	1	1	1	1	1	2
	0	0.5	0.5	336	0	0	4	3	4	3	3	3
	0.5	0	0	84	4	−6	9	9	**10**	9	6	9
	0.5	0	0.5	84	4	−6	8	6	8	6	5	6
	0.5	0.5	0	84	2	−4	6	7	8	6	5	8
	0.5	0.5	0.5	84	4	−3	5	5	6	5	5	5
MAR	0.5	0.5	0	84	2	−7	9	9	10	9	**11**	**10**
	0.5	0.5	0.5	84	4	−7	9	8	9	8	7	7
*Per cent power*												
MCAR	0	0.5	0	336	100	100	100	100	100	100	100	100
	0	0.5	0.5	336	100	**97**	100	100	100	100	100	100
	0.5	0.5	0	84	86	**51**	77	76	76	76	78	77
	0.5	0.5	0.5	84	76	**41**	68	66	69	66	68	67
MAR	0.5	0.5	0	84	86	**43**	72	72	72	73	73	73
	0.5	0.5	0.5	84	76	**36**	64	65	66	64	63	65

Monte Carlo error is ≤0.01 for bias, ≤3 per cent for per cent error in model standard error and ≤1.6 per cent for power. Bold cells have bias greater than 0.03, more than 10 per cent error in model standard error or power more than 3 per cent worse than method T.

## 5. RESULTS FOR THE RENAL CANCER DATA

As stated in Section 2, the analysis model of interest for these data is a proportional hazards model including covariates esr, haem, who, trt, (wcc) ^3 and log (t_mt+1). For ease of comparison, we scale the quantitative covariates esr, haem, (wcc) ^3 and log (t_mt + 1) by their CC standard deviations.

Before imputing the missing values, the skewed variables wcc and t_mt were transformed to an approximate Normal distribution using the lnskew0 program in Stata, which replaces a variable *X* with log(±*X* − *k*) where *k* and the sign are chosen so that log(±*X* −*k*) has zero skewness. Although esr was non-Normally distributed, it was not transformed because exploratory linear regression on the other covariates suggested that its conditional distribution was approximately Normal.

Imputation was performed on the transformed variables using the ice routine in Stata [2, 3] and including the outcome variables appropriate to each method. Transformed values of wcc and t_mt were converted back to the original scale and then formed into the terms wcc^3 and log(t_mt+1) for the analysis model. The COX and COX* methods were implemented by additional programming within ice. We used *m* = 1000 imputations so that Monte Carlo error did not disguise any differences between methods.

We first look at differences between the CC method and all imputation methods (Table VII). One would expect standard errors for the coefficient of a variable *X* to be smaller by MI than by CC when there are a substantial number of observations with observed *X*, but missing data in other variables. In the present data, this would suggest that, compared with CC standard errors, MI standard errors would be similar for esr and smaller for all other variables. The expected pattern is observed for the other variables. However, the standard errors for esr are somewhat increased. This may reflect other features of the current data or may be a chance finding. There are substantial differences in point estimates.

**Table VII:** Renal cancer data: results of proportional hazards models by complete cases and eight different imputation methods. Tabulated values are 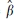 (standard error).

	CC	Imputation methods (*n*=347)						
		
Variable	(*n* = 169)	NO-T	LOGT	T	T2	NA	COX	COX*
esr/35.1	**0.30**	**0.10**	0.24	**0.21**	**0.11**	0.26	0.25	0.25
	(0.11)	(0.10)	(0.12)	(0.12)	(0.12)	(0.12)	(0.12)	(0.11)
who2	**−0.95**	−0.84	−0.87	−0.86	−0.84	−0.87	−0.87	−0.87
	**(0.24**)	(0.17)	(0.17)	(0.17)	(0.17)	(0.18)	(0.17)	(0.17)
who3	−0.62	−0.62	−0.62	−0.61	−0.61	−0.61	−0.61	−0.61
	**(0.22)**	(0.14)	(0.15)	(0.15)	(0.15)	(0.15)	(0.15)	(0.15)
haem/2.00	**−0.44**	**−0.33**	−0.27	**−0.28**	**−0.33**	−0.26	−0.26	−0.26
	(0.12)	(0.09)	(0.10)	(0.10)	(0.10)	(0.10)	(0.10)	(0.10)
wcc^3/13.5	**0.41**	0.33	0.34	0.34	0.33	0.34	0.34	0.34
	**(0.13)**	(0.08)	(0.08)	(0.08)	(0.08)	(0.08)	(0.08)	(0.08)
log(t_mt+1)/1.42	−0.24	−0.23	−0.24	−0.24	−0.23	−0.24	−0.24	−0.24
	**(0.09)**	(0.06)	(0.06)	(0.06)	(0.06)	(0.06)	(0.06)	(0.06)
trt	**−0.44**	−0.37	−0.37	−0.37	−0.37	−0.37	−0.37	−0.37
	**(0.16)**	(0.12)	(0.12)	(0.12)	(0.12)	(0.12)	(0.12)	(0.12)

Bold cells indicate estimates that differ from the NA estimate by more than 20 per cent of the NA standard error, or standard errors that differ from the NA standard error by more than 20 per cent of the NA standard error. Monte Carlo error in parameter estimates is no more than 0.003 in all cases.

Turning to comparisons between imputation methods, the main differences are seen for esr, with T2 and NO-T giving point estimates less than half those for other methods. Smaller differences are seen for other methods. The only other variable whose coefficients show substantial differences between imputation methods is haem. This is the variable with the strongest correlation (−0.61) with e s r, and the differences between the methods reflect residual confounding as a consequence of the attenuated estimates of the coefficient of esr.

## 6. DISCUSSION

We have developed an approximate theoretical rationale for imputing missing covariates in a Cox model using new methods based on the cumulative baseline or marginal hazard (NA, NA-INT, COX and COX*). These methods have the appealing property that they are invariant to monotonic transformation of the time axis, like the Cox proportional hazards model itself, but unlike more commonly used methods (LOGT and T).

Our simulation study allows us to choose between these methods. The NA method performed at least as well as the more complex NA-INT, COX and COX* methods, appearing to have the lowest bias and highest power in most simulations. We therefore consider this to be the best method in general. The NA method is simple to implement in standard software. For example, using ice in Stata [2, 3], after the data have been stset, the Nelson–Aalen estimator is produced by sts gen HT=na and then the MICE algorithm is implemented by ice HT _d X* with appropriate options.

However, all methods were somewhat biased towards the null when covariates were strongly predictive of outcome. This is because the imputation models were not entirely correct. The MI procedure might be improved by using predictive mean matching [19], which aims to draw from the empirical distribution rather than the fitted conditional distribution. Our explorations of this approach in the context of our simulation studies suggest that it can perform very poorly: in particular, when there is no true association between covariate and outcome, predictive mean matching gives implausible distributions of imputed values and very variable estimated coefficients. This appears to be a consequence of small imputation models; strengths and limitations of predictive mean matching are a topic for further research.

This paper did not aim to compare MI with complete cases analysis. The standard view is that MI is more efficient than complete cases for estimating the coefficient of a variable whenever some of the other model covariates are incomplete [11]. Our results support this view, since MI procedures had greater power than complete cases in Table VI but not in Tables III, IV and V. Indeed, MI had worse power in Tables III, IV and V because we used too few imputations: had our aim been a fair comparison of MI with complete cases, we would probably have needed to use *m* = 50 or more imputations.

We assumed a proportional hazards survival model. Other non-parametric survival models, such as the accelerated life model and the proportional odds model, do not yield simple imputation models for the covariates. For the proportional odds model, it can be shown that a Taylor series approximation with β_*X*_ ≈ 0 suggests a logistic model for *X* on *S*_0_(*T*), *D* and their interaction. Thus in principle, different methods are required for these models. We suggest that the NA method might be a reasonable first choice, but that more flexible imputation models should be carefully considered.

We have explored and compared methods in the setting of a single incomplete covariate, but our finding that *D* and *H*_0_(*T*) should be included in imputation models for incomplete covariates is equally relevant for any form of MI that is based on regression models for incomplete covariates. These include imputing from a multivariate normal distribution [13], imputing using monotone missing methods and imputing via chained equations [5]. We therefore recommend that, instead of the logarithm of survival time, imputation should be based on the Nelson–Aalen estimate of the cumulative hazard to the survival time.

## APPENDIX A

Under the PH model *h(t* |*X, Z)* = *h*_0_(*t*)exp(β_*X*_*X* + β_*Z*_*Z*), the log-likelihood for the outcomes, given complete data, is

so by Bayes' theorem, the conditional distribution of *X* given the observed covariates is

(A1)

### A.1. Binary X

Writing logit *p*(*X* = 1|*Z*) = ζ_*Z*_, we have

(A2)

In general, this is not a standard logistic regression because of the exp (β_*Z*_*Z*) term on the right-hand side. However, if there is no *Z*, then we have the exact result that

(A3)

so the correct model for imputing missing *X* is a logistic regression on *D* and *H*_0_(*T*). If in addition β_*X*_ is small, the model further simplifies to logit *p*(*X* = 1|*T, D*) = ζ + β_*X*_(*D* — *H*_0_(*T*)), but this simplification is unlikely to be useful in practice, and we do not pursue it further.

More generally, in the presence of a single categorical *Z*, model (A2) is exactly a logistic regression on *D, Z, H*_0_(*T*) and the interaction between *Z* and *H*_0_(*T*).

In other cases, we have no exact results. However, if we assume an exposure model logit *p*(*X* = 1|*Z*) = ζ_0_ + ζ_1_*Z* and approximate exp(β_*Z*_*Z*)≈exp(β_*Z*_) (where  is the sample mean of *Z*) in (A2) for small var(β_*Z*_*Z*), we get

suggesting imputing missing *X* via logistic regression on *D*, *H*_0_(*T*) and *Z*. A more accurate approximation is exp(β_*Z*_*Z*)≈exp(β_*Z*_){1+β*Z*(*Z* − )}, giving

suggesting imputing missing *X* via logistic regression on *D*, *H*_0_(*T*), *Z* and the interaction *H*_0_(*T*)×*Z*.

### A.2. Normal X

We assume an exposure model *X*|*Z*∼N(ζ_0_ + ζ_1_*Z*,σ^2^), so that equation (A1) gives

(A4)

This is not a Normal (or any other common) distribution because of the exp(β_*X*_*X*) term, so we make approximations for small var(β_*X*_*X* + β_*Z*_*Z*). We first consider a linear approximation  so that (A4) gives

hence approximately

(A5)

implying again a linear regression on *D*, *H*_0_(*T*) and *Z*. A quadratic approximation for e^β_*X*_*X*+β_*Z*_*Z*^ gives

(A6)

hence *X*|*T, D,Z* is approximately Normally distributed with mean

(A7)

and variance

(A8)

This is not linear in the parameters. However, ignoring terms in  gives a linear regression on *D, H*_0_(*T*), *Z* and *H*_0_(*T*)×*Z*. This approximation is valid for small —that is, for small var(β_*X*_*X*) and/or for small *H*_0_(*T*)—but has too large a slope for large *H*_0_(*T*).
